# Predicting the possible effect of miR-203a-3p and miR-29a-3p on *DNMT3B* and *GAS7* genes expression

**DOI:** 10.1515/jib-2021-0016

**Published:** 2021-12-16

**Authors:** Afgar Ali, Sattarzadeh Bardsiri Mahla, Vahidi Reza, Farsinejad Alireza

**Affiliations:** Research Center for Hydatid Disease in Iran, Kerman University of Medical Sciences, Kerman, Iran; Student Research Committee, Faculty of Allied Medicine, Kerman University of Medical Sciences, Kerman, Iran; Department of Hematology and Medical Laboratory Sciences, Faculty of Allied Medicine, Kerman University of Medical Sciences, Kerman, Iran; Cell Therapy and Regenerative Medicine Comprehensive Center, Kerman University of Medical Sciences, Kerman, Iran

**Keywords:** bioinformatics, *DNMT3B* gene, *GAS7* gene, melanoma, microRNAs

## Abstract

Aberrant expression of genes involved in methylation, including DNA methyltransferase 3 Beta (*DNMT3B*), can cause hypermethylation of various tumor suppressor genes. In this regard, various molecular factors such as microRNAs can play a critical role in regulating these methyltransferase enzymes and eventually downstream genes such as growth arrest specific 7 (*GAS7*). Accordingly, in the present study we aimed to predict regulatory effect of miRNAs on *DNMT3B* and *GAS7* genes expression in melanoma cell line. hsa-miR-203a-3p and hsa-miR-29a-3p were predicted and selected using bioinformatics software. The Real-time PCR technique was performed to investigate the regulatory effect of these molecules on the *DNMT3B* and *GAS7* genes expression. Expression analysis of *DNMT3B* gene in A375 cell line showed that there was a significant increase compared to control (*p* value = 0.0015). Analysis of hsa-miR-203a-3p and hsa-miR-29a-3p indicated the insignificant decreased expression in melanoma cell line compared to control (*p* value < 0.05). Compared to control, the expression of GAS7 gene in melanoma cells showed a significant decrease (*p* value = 0.0323). Finally, our findings showed that the decreased expression of hsa-miR-203a-3p and hsa-miR-29a-3p can hypothesize that their aberrant expression caused *DNMT3B* dysfunction, possible methylation of the *GAS7* gene, and ultimately decreased its expression. However, complementary studies are necessary to definite comment.

## 1 Introduction

Skin cancer that is known as the most common form of human cancer, divided into three main types: basal cell carcinoma, squamous cell carcinoma, and melanoma. Melanoma is a malignant tumor of melanocytes (special cells that produce melanin pigments) that despite its low prevalence (5%), has the highest mortality compared to other types (75%). On the other hand, the high-rate invasion of melanoma to other tissues is a considerable factor for early diagnosis and treatment of this disease. Several factors such as exposure to ultraviolet light (through DNA mutations), severe sunburn, immunodeficiency, and genetic predisposition make people more susceptible to melanoma [1–4].

Researches show that about 5–10% of melanoma cases are people who have genetic alterations or mutation in several genes including CDKN2A (Cyclin Dependent Kinase Inhibitor 2A), CDK4 (Cyclin-dependent kinase 4), BAP1 (BRCA1 associated protein-1), POT1 (Protection Of Telomeres 1), ACD (Adrenocortical dysplasia), TERF2IP (Telomeric repeat-binding factor 2-interacting protein 1), and TERT (Telomerase reverse transcriptase) that lead to the tumorgenesis [5]. Recently, it is suggested that in addition to genetic alterations, changes in epigenetic pattern (abnormal promoter methylation, histone modifications, and dysexpression of small non-coding RNAs) also play a critical role in tumor formation [6, 7]. In mammals, methylation occurs mainly in CpG islands that are located in promoter, untranslated, and exonic regions of genes and through DNA methyltransferases (*DNMT1*, *DNMT3A* and *DNMT3B*). Recent studies indicate that abnormal methylation (increase or decrease) of CpG islands may result in tumorigenesis, so that increased methylation in the regulatory regions of tumor suppressor and DNA repair genes silences them and leads to cancer progression [8]. Abnormal DNA methylation is considered as an important epigenetic marker in melanoma pathogenesis [9] and dysexpression of methyltransferases such as *DNMT3B*, can alter methylation pattern in genes promoter [10, 11]. One of the important regulators of *DNMT3B* are small non-coding molecules called microRNAs, that are able to bind to the UTR regions of the target genes (through their "seed" region with 2–8 nucleotides) and act as post-transcriptional modification regulators. Evidences have shown that microRNA expression patterns for each cancer can be different and therefore used as a clinical prognostic factor [12, 13]. Dysregulation of these molecules can alter the expression pattern of DNA methyltransferases such as *DNMT3B* and lead to abnormal methylation process. There are many genes that their expression affected by improper methylation pattern; for example, growth arrest-specific genes (*GAS* gene family) that organize several biological functions including microfilament organization, neuronal differentiation, apoptosis, and cell cycle regulation. *GAS7* which is located at 17P13.1, mainly expressed in differentiated brain cells and cerebellar purkinje neurons and its product i.e., *GAS7* protein, plays an important role in nervous system development. In addition, this gene also has tumor suppressor activity [14, 15]. According to this information, dysfunction of microRNAs can play an important role in the development of cancer, such as melanoma [12]. Therefore, in the present study, we aimed to predict microRNAs involved in *DNMT3B* gene methylation through bioinformatics approaches and examined their effect on *DNMT3B* and *GAS7* genes expression in melanoma cells.

## 2 Materials and methods

### 2.1 MicroRNA and target mRNA prediction

Because of overexpression of *DNMT3B* gene in many tumor cells, it was selected and subsequently analyzed using DianamT, miRanda, miRDB, miRWalk, RNAhybrid, PICTAR4, PICTAR5, PITA, RNA22, and Targetscan through the miRWalk 2.0 database hyperlink (http://zmf.umm.uni-heidelberg.de/apps/zmf/mirwalk/predictedmirnagene.html). According to the algorithm of the mentioned programs, a large number of miRNAs were predicted based on the following criteria: the longest seed region, conserved pairing of seed region, and the number of software that simultaneously predicted the target miRNAs. Finally, two microRNAs were selected based on the mentioned items for the *DNMT3B* gene binding.

### 2.2 Stem-loop, primers, and special probes design

**Figure 1:**
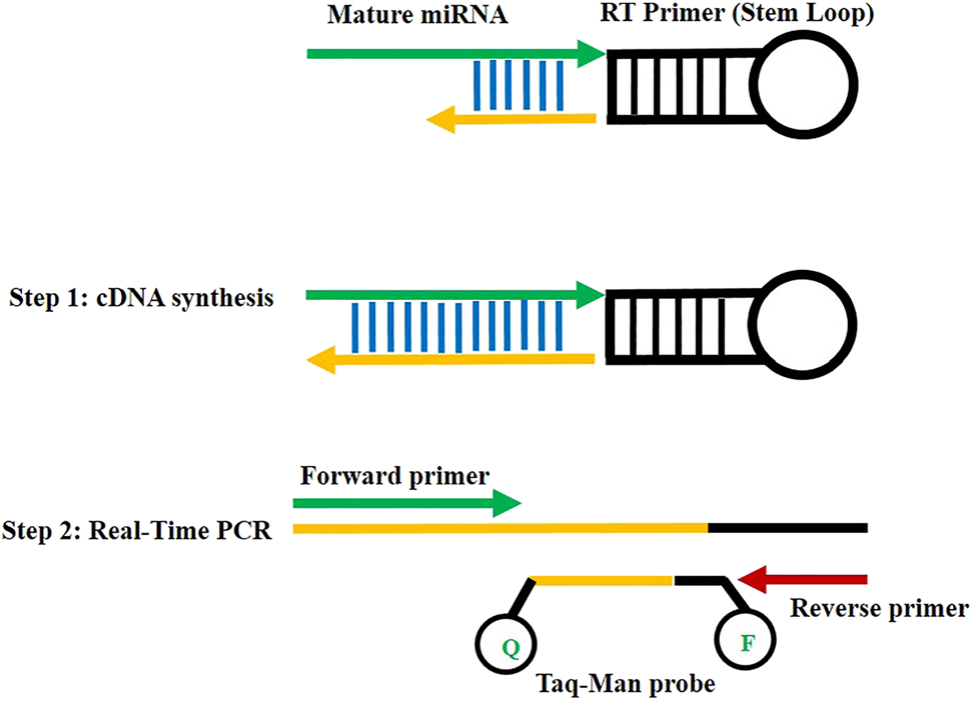
Schematic representation of stem loop structure and miRNAs amplification using the stem loop RT approach. This method consists of two steps: 1 – stem loop RT amplification (cDNA synthesis) 2 – real-time PCR reaction. In this method, first six nucleotides of 3′ of miRNA target are reversed, complemented, and attached to 3′ position of stem loop RT and cDNA is synthesized by reverse transcriptase. The synthesized product (cDNA) is then amplified by real-time PCR and universal TaqMan probe.

The sequences of predicted microRNAs were retrieved from the microRNA database (http://www.mirbase.org/). These target microRNAs included hsa-miR-203a-3p and hsa-miR-29a-3p. In order to detection of the minimum amount of target microRNAs and increase the necessary sensitivity, we used the stem loop sequence from Faridi et al. publication [16]. The designed miRNA could detect the last six nucleotides at the end of stem-loop which complement the 3′ region of the each mature miRNA (Figure 1). To design forward primers, almost all mature microRNAs sequences were used. Then, the melting temperature (*T*_m_) of the primers and probes and also secondary structure of all sequences was adjusted using Gene Runner software and mFold web server (http://mfold.rna.albany.edu/?q=mfold/) (https://bio.tools/mfold). To determine the specificity of each primer, the blast primer analysis was performed using the Primer-Blast (https://www.ncbi.nlm.nih.gov/tools/primer-blast). Then, the experimental specificity of each microRNA was examined and confirmed by amplification sequence analysis (Real-time PCR). The sequences of designed reverse transcriptase (RT) stem-loops, primers and probe are presented in Table 1.

**Table 1:** Designed RT stem-loops, primers and probe.

miRNA	Accession number	Designed sequence
RT primer miR-203a-3p	MIMAT0031890	GTATGCTGCTACCTCGGACCCTGCTTAGTGCCATGCCTGCCATCGAGCAGCATAC AACTGT
RT primer miR-29a-3p	MIMAT0004503	GTATGCTGCTACCTCGGACCCTGCTTAGTGCCATGCCTGCCATCGAGCAGCATAC CTGAAC
RT primer U6	NR_004394.1	GTATGCTGCTACCTCGGACCCTGCTTAGTGCCATGCCTGCCATCGAGCAGCATAC CGAATT
F-miR-203a-3p	MIMAT0031890	AGTGGTTCTTAACAGTTCAACAGTT
F-miR-29a-3p	MIMAT0004503	GCGTGATTTCTTTTGGTGTTCAG
F-U6	NR_004394.1	GCAAGGATGACACGCAAATTCG

Taq man probe sequence: FAM 5′AGTGCCATGCCTGCCATCGAGC 3′ BHQ-1, universal reverse primer sequence: GCTGCTACCTCGGACCCT, F: forward primer.

### 2.3 Cell preparation

A375 melanoma cell line (ATCC^®^ CRL-1619™) and normal melanocytes (ATCC^®^ PCS-200-013™) were purchased from the cell bank of Pasteur Institute of Iran (IPI), Tehran, Iran. The cells were cultured in Dulbecco’s minimum Eagle’s medium (DMEM) with 10% FBS, 2 mM glutathione, 100 U/mL of penicillin and 100 μg/mL of streptomycin. Transfer to fresh media was performed when confluency was around 90% and finally, cells were harvested using 0.25% trypsin-EDTA.

### 2.4 Experimental design

In relation to experimental design, gene expression was performed by real-time PCR with three biological and two technical replicates. In this case, three separate samples were prepared from each cell line and then RNA extraction and cDNA synthesis were performed for each sample. Finally, the gene expression in each sample was evaluated using real-time PCR and in duplicate.

### 2.5 Extraction of target microRNAs and RNAs

Extraction of target microRNAs was carried out with a slight modification of the RNA extraction protocol of “Sinaclone RNX-Plus Solution for total RNA isolation”. Briefly, 1 mL of RNX- Plus extraction solution was added to 5–6 × 10^6^ cells. The cells were incubated for 3 min, then 200 μL of 1-bromo-3-chloropropane solution added to each tube and centrifuged at 4 °C (12,000*g* for 25 min). The supernatant was separated and transferred to a new sterile tube and the previous step was repeated with 100 μL of 1-bromo-3-chloropropane. Finally, the aqueous phase was transferred to another tube and the equal volume of isopropanol was added. The tubes were kept overnight at −20 °C (20 min for total RNA) and then centrifuged at 4 °C (12,000*g* for 1 h). After centrifugation, the supernatant was discarded and 1 mL of 70% ethanol was added and centrifuged at 12,000*g* for 30 min. The supernatant was discarded again and tubes were kept upside down at room temperature. At the end, 50 μL of DEPC-Treated Water and 5 units of RNase free-DNase I enzyme were added to each tube, incubated for 10 min at room temperature, and then inactivated for 5 min at 80 °C. The concentration and purity of the extracted miRNAs and total RNA were determined and finally, all samples were stored at −70 °C until analysis.

### 2.6 cDNA synthesis and real-time PCR

Following RNA extraction, 1000 ng of each sample was reversed using Mu-MLV reverse transcriptase according to the Thermo Scientific™ RevertAid RT Reverse Transcription Kit protocol. The cDNA was stored at −70 °C until analysis. The qPCR reaction was performed on a mixture of 12.5 μL of SYBR™ Green PCR Master Mix, 0.1 μM of probe, 0.2 μM of each oligonucleotide primers and 2 μL of cDNA. Real-time PCR reaction occurred under these conditions: initial denaturation at 95 °C for 40s, followed by 45 cycles consist of denaturation at 95 °C for 40s, annealing at 60 °C for 20s, and final expansion at 72 °C for 35s. The β-actin gene was used as the internal reference gene. Finally, PCR efficiency and expression of each mRNA were assessed using linreg software (LinRegPCR (11.0) – Gene Quantification). A *p* value < 0.05 was considered statistically significant.

### 2.7 MicroRNA cDNA synthesis and real-time PCR

Following miRNA extraction, cDNA was synthesized using Mu-MLV reverse transcriptase. Four μL of extracted miRNA (adjusted for 2000 ng miRNA) was added to 1.5 μL of stem-loop (1.100 dilution of 100 μM stock) and 5 μL of double distilled water. This mixture was incubated for 5 min at 65 °C in a thermocycler. Immediately, 2 μL of dNTP (10 mM), 4 μL of 4× buffer, and 0.5 μL of RNase inhibitor (20 units), 2 μL of DTT (10 mM), and 1 μL of reverse transcription enzyme (50 μL) were added to this mixture. Synthesis of cDNA was performed for 1 h at 44 °C and 5 min at 85 °C for enzyme inactivation. The synthesized cDNA was stored at −20 °C until Real-time analysis. Real-time PCR was performed using specific forward primer and probe for each microRNA. The final mixture was consisting of 6.25 μL of 2× qPCR Master Mix, 0.7 μM of reverse primer, 0.5 μM of forward primer and 0.2 μM of specific probe in a total volume of 12.5 μL. The enzyme was initially activated at 95 °C for 30s, followed by 45 cycles consist of 95 °C for 15s and 60 °C for 45s. The U6 gene was selected as the internal reference gene. Statistical analysis was carried out for the relative expression of each microRNA using Pfaffl method in GraphPad Prism 9.1.2 software. A *p* value < 0.05 was considered statistically significant.

## 3 Results

### 3.1 Predicting of miRNAs and target mRNAs, and designing of stem-loops, primers, and probes

**Figure 2:**
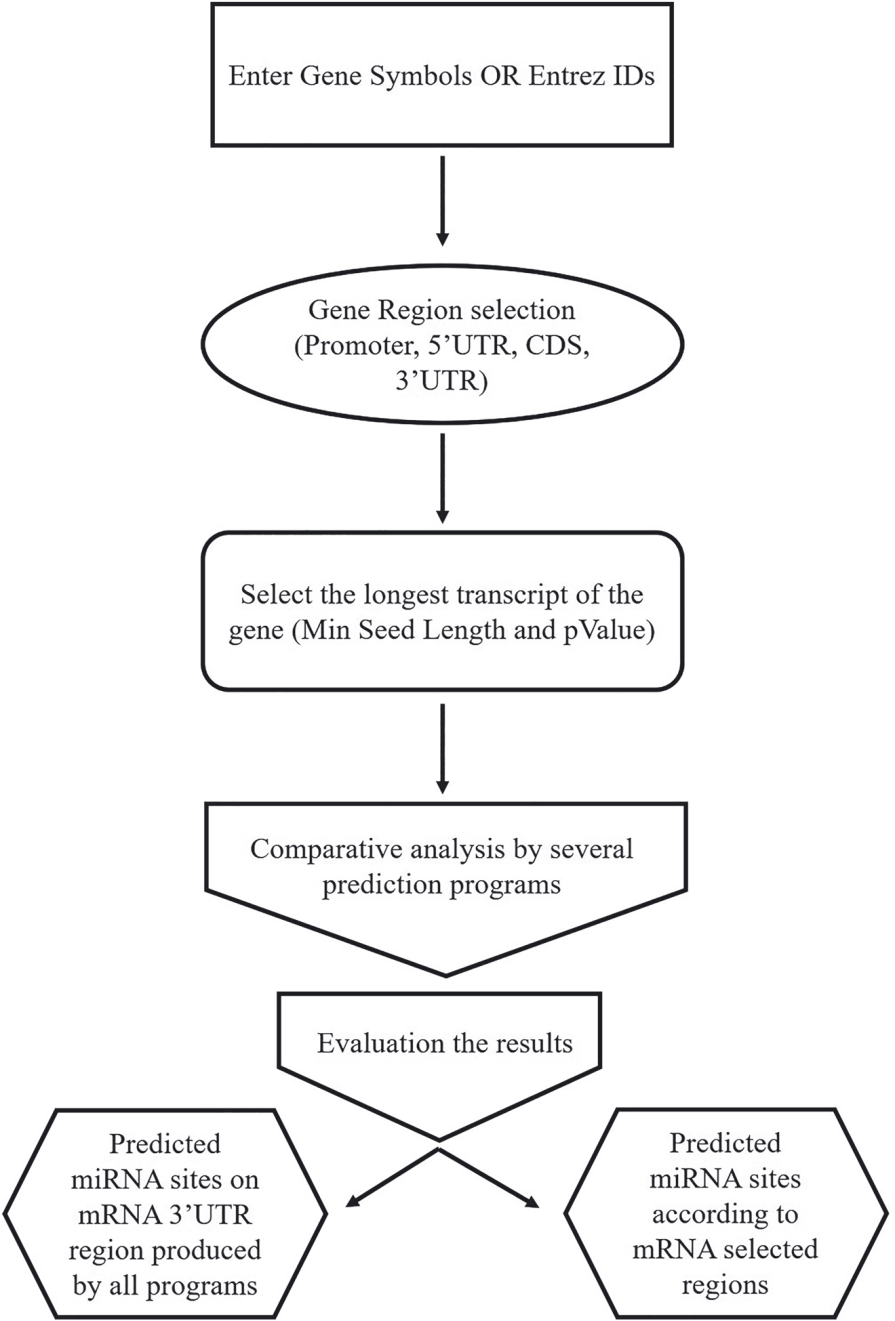
An illustration of the workflow that was performed in this study using mirwalk software. Also, this workflow was carried out for the majority of the software mentioned in this study, but with a few changes.

Gene repression pathways were analyzed using various popular algorithms including miRanda, miRDB, miRWalk, RNAhybrid, PICTAR4, PICTAR5, PITA, RNA22, and Targetscan to predict the miRNAs targets including 3^’^UTR region of *DNMT3B* mRNA. More than 20 miRNAs targeting *DNMT3B* gene were identified and confirmed by six miRNA prediction algorithms. As mentioned in the Materials and Methods, three criteria were applied for selection of miRNAs; the longest seed region, conserved pairing of seed region, and the number of software that simultaneously predicted the target miRNAs. According to these criteria, 2 miRNAs were selected for future study (Figure 2, Tables 2 and 3).

**Table 2:** Prediction of target miRNAs based on the number of algorithms.

Gene name	MicroRNA	D	miRa	miRD	miRW	RNAh	PIC4	PIC5	P	R22	Ts	Sum
DNMT3B	hsa-miR-203a-3p	1	1	0	1	1	0	1	0	0	1	6
	hsa-miR-29a-3p	1	1	1	1	1	0	0	0	0	1	6

D: DIANAmT, miRa: miRanda, miRD: miRDB, miRW: miRWalk, RNAh: RNAhybrid, PIC4: PICTAR4, PIC5: PICTAR5, P: PITA, R22: RNA22, Ts: target scan.

**Table 3:** Prediction of target miRNAs based on seed length and gene region.

Gene name	RefSeq ID	MicroRNA	Seed length	3′UTR length	Region
*DNMT3B*	NM_006892	hsa-miR-29a-3p	8	1470	3′UTR
	NM_006892	hsa-miR-203a-3p	7	1470	3′UTR

Specific stem-loop RT for each miRNA was designed by adding six complementary nucleotides to the 3^’^UTR ends of the *DNMT3B* gene. Forward primers were designed alongside the reverse primer and the TaqMan probe for qPCR. NCBI Primer-BLAST for characterization of each miRNA showed that no sequence except corresponding miRNA binds to the target sequence. Characterization results showed 100% specificity for each miRNA.

### 3.2 Expression analysis of the predicted miRNAs, *DNMT3B*, and *GAS7*:

**Figure 3:**
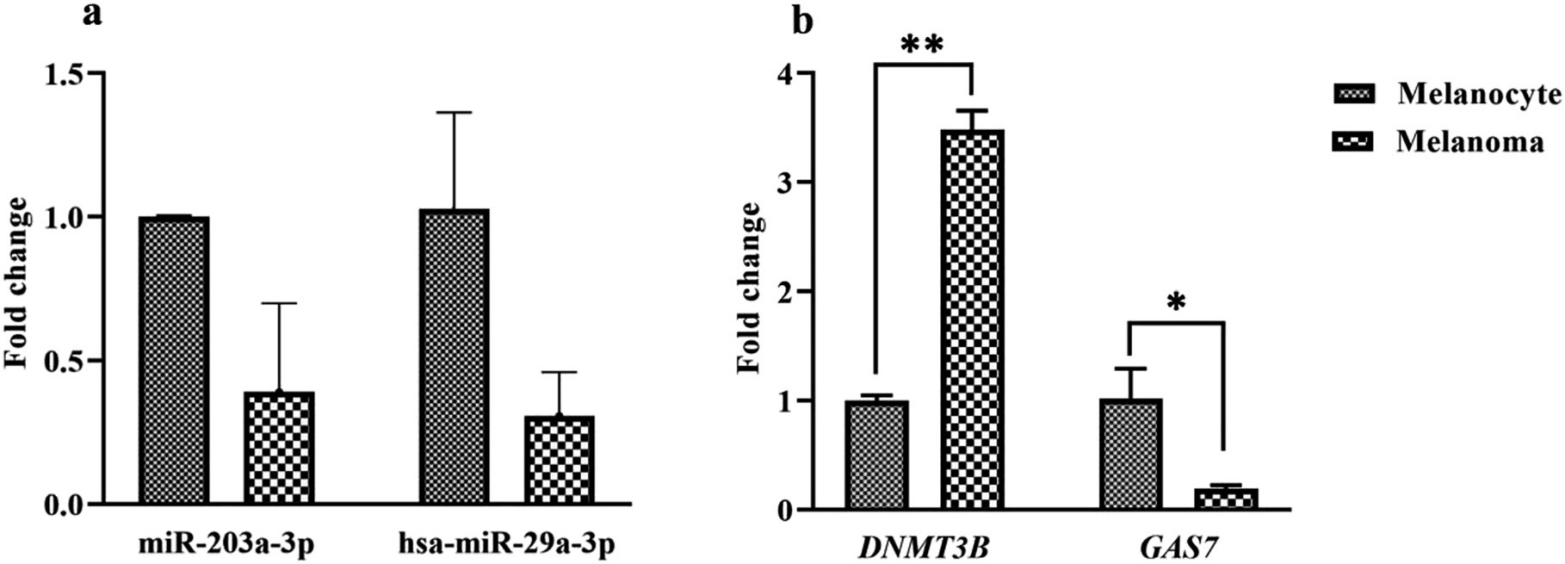
The expression of hsa-miR-203a-3p, hsa-miR-29a-3p, and target genes (*DNMT3B* and *GAS7*) in melanocyte and melanoma cells. As illustrated, unlike miRNAs, the expression of target genes were significantly changed in melanoma cells; *DNMT3B* was up- and *GAS7* was down-regulated, respectively. ^*^p < 0.05 and ^**^p < 0.01.

In the A375 melanoma cell line, two miRNAs (hsa-miR-203a-3p and hsa-miR-29a-3p) were selected from several microRNAs predicted by different software algorithms. As illustrated in Figure 3a, the expression of two selected microRNAs was decreased, insignificantly (*p* < 0.05). On the other hand, the *DNMT3B* gene was significantly upregulated in melanoma cells compared to control cells (*p* = 0.0015, Figure 3b). Compared to normal cells, the expression analysis evidenced that *GAS7* gene was significantly downregulated in the A375 cells (*p* = 0.0323, Figure 3b). These findings raise the possibility of methylation of *GAS7* gene following overexpression of *DNMT3B* gene.

## 8 Discussion

Epigenetic changes such as aberrant hypermethylation, by inducing post-transcriptional gene silencing, may contribute to inactivation of tumor suppressor genes and cancer pathogenesis [17–19]. One of the proposed mechanisms for aberrant methylation in promoters of tumor suppressor genes is the improper regulation and expression of *DNMT* genes [20]. Among all DNMTs, *DNMT3B* plays a major pro-tumorigenic role in human melanoma [11]. In several studies, it has been shown that there is a significant relation between aberrant expression of tumor suppressors and increased expression of *DNMT3B* gene [21, 22].

One of the important regulators of *DNMT3B* is small non-coding molecules called microRNAs [12]. Computational methods play an important role in predicting and finding miRNA targets with several designed algorithms [23]. Among these methods, high-throughput techniques such as next-generation sequencing and microarray have a high ability for determining expression profile/quantification of a large number of miRNAs. These methods are costly and not easily accessible [24]. On the other hand, the short length of some sequences such as miRNA also limits these methods [25]. As an alternative, in this study, we used bioinformatics prediction to find miRNAs that are involved in the regulation of *DNMT3B* and *GAS7* genes of melanoma cells and melanocytes. In the following, we performed stem-loop technique [26], which is highly accurate and cost-effective method (due to use of universal oligonucleotides), to investigate the expression of the predicted microRNAs (hsa-miR-203a-3p and hsa-miR-29a-3p) in melanoma cells.

Regarding miRNAs prediction, it should be noted that the ability of software is different based on the defined algorithms. For example, according to the results obtained from the used prediction software for hsa miR-29a-3p, RNA22 and PICTAR4 software were not able to detect this miRNA. On the other hand, PITA software, essentially detects microRNAs by the amount of access to sites within the target mRNA. It first evaluates the 3′UTR of important target positions, through searching the pairs of near-perfect connections, and then calculates G for each position (G is actually a score for the probability of a target site reacting with miRNA). Considering upstream and downstream target positions for G is optional. Nevertheless, this software could not succeed in prediction hsa miR-29a-3p.

On the other hand, the miRwalk algorithm detects the sequence between miRNA and the target gene sequence, based on the longest sequences. According to the Watson-Creek complement pair base, the work of the software begins with searching in the complete gene sequence as well as the mitochondrial genome and then finding a region with at least seven matched nucleotides with the target microRNA. Next, the longest seed sequences that situated at four protein-coding regions (promoter, 5′UTR, CDS, and 3′UTR) are identified. Using different algorithmic approaches helped us to identify and predict the best miRNAs for future evaluations. The findings exhibited that hsa-miR-203a-3p and hsa-miR-29a-3p are bioinformatically able to bind to the 3^’^UTR region of the *DNMT3B* gene and play a possible role in its regulation. To investigate the possible indirect effect of miRNAs on expression of *DNMT3B* and *GAS7* genes, we quantified the expression of these genes in melanoma cells and normal melanocytes. The results demonstrated that the *DNMT3B* and *GAS7* genes were significantly up- and down-regulated in melanoma cells compared to melanocytes, respectively (Figure 3b). Decreased expression of *GAS7* gene in melanoma cells can be attributed to increased expression of genes involved in methylation (*DNMT3B*). Interestingly, Ashktorab et al. in a study on colorectal neoplasia showed that there were 13 significant methylated genes in 355 CpG islands (14 islands were in promoter regions). Among the most methylated genes were *ATXN7L1*, *BMP3*, *EID3*, *GPR75*, and especially *GAS7* [27, 28].

Although not significant, it seems that a 0.390-fold reduction in expression of miR-203a-3p and a 0.307-fold reduction in miR-29a-3p expression (Figure 3a) could have direct and indirect regulatory effects on *DNMT3B* and *GAS7* genes, respectively. The potential regulatory effect of miRNAs on hypermethylation of tumor suppressor genes has been confirmed in other studies [29, 30]. On the other hand, Micevic and colleagues evidenced that induction of miR-29a-3p in acute myeloid leukemia and Burkitt’s lymphoma could suppress *DNMT3B* and subsequently cause promoter hypomethylation of tumor suppressor genes [11].

Overall, our findings suggest that epigenetic matters, particularly methylation, have a significant effect on tumorigenesis and should be considered as one of the vital issues in melanoma. It should be noted that this claim is based only on bioinformatics and gene expression findings; therefore, its final confirmation requires further epigenetic studies such as methylation and acetylation. Also, the expression of gene can be influenced by several miRNAs and each miRNA also may be targeted several genes [31, 32]. Therefore, in order to make a definite comment about the pathogenesis of melanoma and also factors involved in the insignificant reduction of the studied miRNAs expression, it is necessary to conduct more extensive studies on the other genes, miRNAs, and epigenetic processes such as acetylation.

In conclusion, it seems that our predicted miRNAs have a regulatory role on *DNMT3B* and *GAS7* genes and alteration of their expression can be considered as a new therapeutic approach (alone or in combination with other methods) for melanoma.

## Supplementary Material

Supplementary Material Details

Supplementary Material Details
